# Rapid respiratory panel test for non-COVID-19 pathogen examinations among frontline medical personnel in Taiwan

**DOI:** 10.1186/s12199-021-00945-1

**Published:** 2021-02-13

**Authors:** Yu-Chih Chen, Huei-Wen Lai, I-Lun Hou, Pei-You Hsieh, Po-Yu Wang, Ting-Yuan Ni, Chu-Chung Chou, Yan-Ren Lin

**Affiliations:** 1 Department of Emergency and Critical Care Medicine, Changhua Christian Hospital, Changhua City, Taiwan; 2 Center of Infection Prevention and Control, Changhua Christian Hospital, Changhua City, Taiwan; 3 Department of Pediatric Emergency, Changhua Christian Children’s Hospital, Changhua City, Taiwan; 4 School of Medicine, Kaohsiung Medical University, Kaohsiung, Taiwan; 5 School of Medicine, Chung Shan Medical University, Taichung, Taiwan

To the Editor:

Handling over 100 million COVID-19 cases, frontline medical personnel are threatened due to the high risk of cross-infection. Several studies have recommended that medical personnel with suspicious symptoms (including fever, cough, diarrhea, muscle pain, and loss of smell) clearly receive SARS-CoV-2 testing and at least 14 days of quarantine (close contact with COVID-19 patients without appropriate infection prevention) [1]. Lacking personnel would slow down the hospital operation and further impact patient safety. Notably, we agree that COVID-19 should be excluded first. However, early identification of “non-COVID-19” pathogens would also be beneficial for adjusting the length of quarantine and the policy of workforce resupply. For example, medical personnel with rhinovirus infection might not need 14-day quarantine. Unfortunately, information regarding non-COVID-19 pathogens (including coinfections) among frontline medical personnel is not well known, and we aim to present our experience in Taiwan.

From 1 March to 30 June 2020, a total of 1272 patients were reported to the Taiwan CDC for testing COVID-19 (SARS-CoV-2) in our hospital. Among them, 115 (9%) were frontline medical personnel (handling or facing patients). In addition, 105 of them (91.3%) received rapid respiratory panel test (BIOFIRE® FILMARRAY® Respiratory Panels) in the emergency department (ED) (Table 1). All of them were negative for COVID-19. However, 26 (24.7%) of them tested positive for non-COVID pathogens, including 18 (17.1%) who were positive for human rhinovirus/enterovirus RNA, 2 (1.9 %) who were positive for coronavirus OC43 RNA, and 2 (1.9%) who were positive for coronavirus NL63 RNA (Table 2). Three (2.9%) patients had coinfections (2 or > 2 categories of virus). The first was coinfected with coronavirus OC43 RNA and human rhinovirus/enterovirus RNA, the second was coinfected with adenovirus DNA and human rhinovirus/enterovirus RNA, and the last was coinfected with parainfluenza virus 4 RNA and respiratory syncytial virus RNA.

**Table 1:** Demographics of patients who received non-COVID-19 pathogen examinations

	Medical personnel (*n* = 105)
**Sex**	
Male	19 (18.1%)
Female	86 (81.9%)
Age (years)	
< 31	43 (41.0%)
31–40	40 (38.1%)
41–50	18 (17.1%)
51–60	4 (3.8%)

**Table 2:** Categories of pathogens of 105 patients who received non-COVID-19 pathogen examinations

Detected virus	No. (%)
Rhinovirus/enterovirus RNA	18 (17.1%)
Coronavirus OC43 RNA	2 (1.9%)
Coronavirus NL63 RNA	2 (1.9%)
Adenovirus DNA	2 (1.9%)
Parainfluenza virus 4	2 (1.9%)
Respiratory syncytial virus RNA	1 (1.0%)
Coronavirus HKU1 RNA	1 (1.0%)

Among the medical personnel (with suspected symptoms), our results demonstrated that 24.7% tested positive for non-COVID pathogens. Rhinoviruses and enteroviruses were the leading non-COVID-19 pathogens during the pandemic period. When facing workforce insufficiency, long-term quarantine for medical personnel might not be necessary when their COVID-19 and non-COVID-19 pathogens are both confirmed early. In one testing model, the chance of post-quarantine transmission might obviously decrease after 7 days of quarantine [2]. A rapid respiratory panel test in the ED might be effective for early detection. Finally, we recommend that the quarantine period should be at least 7 days for (suspected symptoms) medical personnel who are negative for all pathogens (including COVID-19 and FILMARRAY Respiratory Panels).

## Data Availability

Not applicable.
